# Pediatric Extraspinal Sacrococcygeal Ependymoma: A Structured Narrative Review of Published Cases and Diagnostic Challenges

**DOI:** 10.3390/cancers18213459

**Published:** 2026-10-26

**Authors:** Eleonora Nativi, Vittorio Maria Virgone, Elisa Bassan, Bianca Stefani, Chiara Cordola, Angela Pucci, Marco Ghionzoli

**Affiliations:** 1 Pediatric Surgery Unit, Maternity and Children Department, University of Pisa, 56126 Pisa, Italy; 2 Division of Pediatric Surgery, Department of Medical Sciences, Surgical Sciences and Neurosciences, University of Siena, 53100 Siena, Italy; 3 Histopathology Department, Pisa University Hospital, 56126 Pisa, Italy

**Keywords:** extraspinal ependymoma, sacrococcygeal ependymoma, pediatric surgery

## Abstract

Background: Extraspinal sacrococcygeal ependymoma is a rare pediatric neoplasm that frequently presents as a superficial sacrococcygeal mass, closely resembling benign conditions such as pilonidal disease or cysts. As a result, preoperative diagnosis is often incorrect, leading to incomplete surgical excision and delayed clinical management. We conducted a comprehensive descriptive review of the pediatric literature to examine its epidemiology, clinical presentation, diagnostic challenges, pathological characteristics, treatment strategies, and outcomes. Materials and Methods: A structured literature search of PubMed, Scopus, and Web of Science from database inception to June 2026 was conducted. Only English studies reporting histologically confirmed extraspinal sacrococcygeal ependymoma in patients younger than 18 years were included. Discussion: Histologically, the vast majority of extraspinal sacrococcygeal ependymomas are of the myxopapillary subtype. Historically classified as grade 1 tumors by the World Health Organization (WHO), they were reclassified as WHO grade 2 in the 2021 WHO Classification of Tumors of the Central Nervous System. This change reflects the rising recognition of the clinically relevant risk of local recurrence and metastatic dissemination of these tumors, despite a relatively indolent growth pattern. Because extraspinal sacrococcygeal ependymomas may recur many years after initial treatment and occasionally metastasize to lymph nodes, lungs, liver, bone, or other distant sites, they should not be considered entirely benign lesions. Then, their management requires an oncological approach despite their often innocuous clinical appearance. Conclusions: Given the risk of delayed recurrence and distant metastasis, long-term follow-up is mandatory. Increased awareness of this singular entity, appropriate preoperative imaging of atypical sacrococcygeal lesions, and multidisciplinary management are essential to optimize outcomes. Future multicenter collaborative studies are needed to establish evidence-based recommendations for diagnosis, treatment, and surveillance.

## Simple Summary

Extraspinal sacrococcygeal ependymoma is a rare tumor in children and adolescents, often presenting as a swelling in the sacrococcygeal region with slow growth and closely resembling common benign conditions such as pilonidal sinus disease or other cystic lesions. As a result, diagnosis may be delayed, and surgical treatment may be initially inadequate. We present a structured narrative review of the pediatric literature to summarize clinical presentation, diagnostic challenges, treatment approaches and, outcomes associated with this uncommon entity. Our objective is to increase practitioner awareness, thereby improving early recognition, appropriate management, and ultimately supporting better care for young patients.

## 1. Introduction

Sacrococcygeal masses in children include a wide spectrum of congenital, inflammatory, and neoplastic conditions. Although the majority of these lesions are benign, their overlapping clinical presentation may pose significant diagnostic challenges and occasionally conceal malignant tumors.

These entities may arise from neural, vascular, lymphatic, or mesenchymal tissues and may represent either isolated lesions or manifestations of a systemic disease [1]. Clinical presentation depends on both the location and size of the lesion and may include low back or sacral pain, abdominal pain, bowel or urinary dysfunction, a palpable pelvic or abdominal mass, anal discharge or bleeding. However, many patients remain asymptomatic [1].

Owing to these nonspecific manifestations and the broad differential diagnosis (Table 1), imaging has a central role in lesion detection, characterization, and surgical planning [1].

Among neoplastic lesions arising in the sacrococcygeal region [3], sacrococcygeal teratoma is the most common entity in childhood, whereas other tumors are seldom encountered but should always be considered in the differential diagnosis of atypical or persistent sacrococcygeal masses. Failure to recognize them may lead to incomplete surgical treatment and delayed oncologic evaluation.

In regard to ependymomas, these account for approximately 9% of all pediatric central nervous system tumors and represent the most common intramedullary spinal neoplasm in this age group [4,5]. Extraspinal localization is exceptionally uncommon, accounting for less than 5% of all ependymomas [6,7,8]. In pediatric patients, the subcutaneous sacrococcygeal region is the most frequent extraspinal site, whereas presacral localization is less common [6,9,10]. Lesions located posterior to the sacrum typically present as asymptomatic or mildly painful subcutaneous masses [8], often closely resembling other benign conditions. Consequently, the diagnosis may only become apparent following surgical excision and definite histological diagnosis.

Due to the uniqueness of pediatric extraspinal sacrococcygeal ependymoma (ESE), current knowledge is based almost exclusively on isolated case reports and small case series, which limits the development of evidence-based diagnostic and therapeutic recommendations. Therefore, we performed a comprehensive review of the pediatric literature to summarize the current knowledge on ESE in children, with particular emphasis on diagnostic pitfalls and clinical management.

## 2. Materials and Methods

A comprehensive literature review was conducted to identify all published pediatric cases of extraspinal sacrococcygeal ependymoma: given the heterogeneous and descriptive nature of the published data, consisting entirely of isolated case reports and small observational series, a structured narrative approach was selected as the most appropriate methodology to summarize the available evidence regarding epidemiology, clinical presentation, diagnostic evaluation, pathological features, treatment strategies, and outcomes.

To ensure a thorough retrieval of published cases, we performed a comprehensive search of the PubMed, Scopus, and Web of Science databases from database inception to June 2026. The search strategy combined terms related to tumor type (“ependymoma”, “myxopapillary ependymoma”), anatomical location (“extraspinal”, “extraneural”, “subcutaneous”, “soft tissue”, “sacrococcygeal”, “coccygeal”, “presacral”), and target age demographic (“child”, “children”, “pediatric”, and “adolescent”).

We focused on studies that reported histologically confirmed ESE in patients younger than 18 years of age, published in English. Articles describing intracranial or intraspinal ependymomas, metastatic lesions to the sacrococcygeal region, or tumors arising primarily within the spinal canal were excluded. In case of duplicate publications describing the same patient, only the most complete and detailed report was included.

For each eligible patient, relevant clinical data, such as sex, age, presenting symptoms, initial clinical diagnosis, treatment, use of adjuvant therapy, recurrence, metastatic disease, and duration of follow-up, were extracted. The extracted data were synthesized with particular emphasis on diagnostic pitfalls, oncologic management, recurrence patterns, and long-term surveillance.

## 3. Results

### 3.1. Literature Search Results

**Figure 1:**
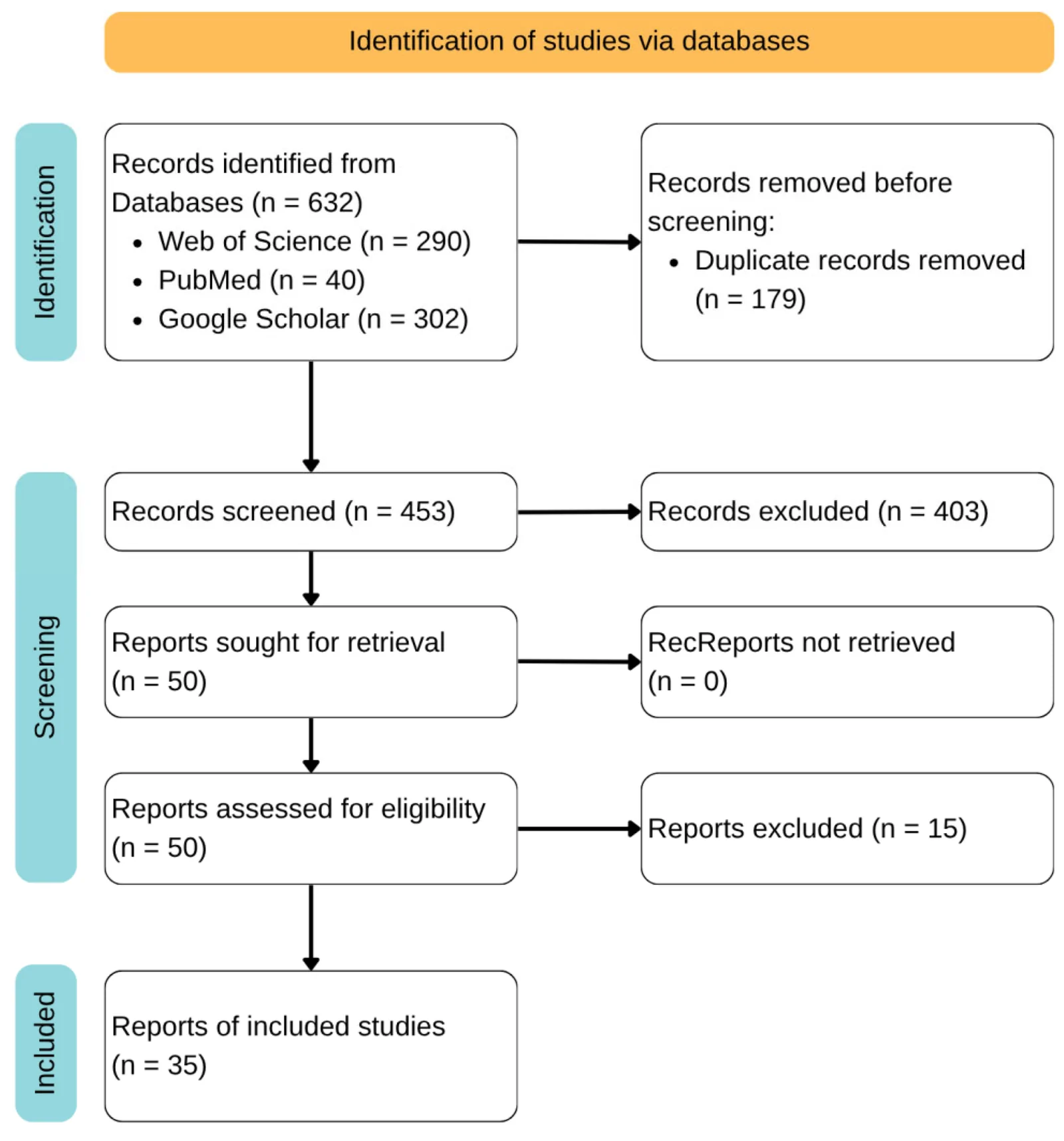
PRISMA 2020 [11] flow diagram illustrating the study selection process.

The literature search identified 632 records (290 from Web of Science, 40 from PubMed, and 302 from Scopus). After removal of duplicate records, 453 unique articles remained for screening. Following title screening, 179 articles were selected for abstract review, of which 50 studies underwent full-text assessment for eligibility. Ultimately, after discarding those papers that, upon closer inspection, did not meet the inclusion criteria, 35 studies were included. See Figure 1 for the PRISMA 2020 Flow Chart.

### 3.2. Published Pediatric Cases

To present a comprehensive overview of the pediatric experience with this tumor, we reviewed all published cases of ESE in patients younger than 18 years and summarized their demographic, clinical, and therapeutic characteristics (see Table 2).

A total of 42 pediatric cases were identified in the literature. The median age at diagnosis was 8 years, with a slight female predominance (21 females, 20 males, 1 not specified). Metastases at surgery were recorded in 4 patients (9.5%). Complete surgical excision was the primary treatment in all cases, while coccygectomy was performed in 13 patients (31%). Among these patients, 11 (84.6%) remained recurrence-free.

Seven recurrences (17%) were reported at a median follow-up of 53 months.

The most frequent preoperative diagnosis was pilonidal cyst, reported in 9 patients (21%), showing the typical superficial location and benign clinical appearance of these tumors. See Table 3 for details on initial misdiagnoses.

### 3.3. Epidemiology

Ependymoma represents the most common tumor of the central nervous system in childhood after medulloblastomas and astrocytomas. It derives from ependymal cells lining the cerebral ventricles and the canal of the spinal cord.

Extraspinal sacrococcygeal ependymoma represents an exceptionally uncommon neoplasm, accounting for less than 5% of all ependymomas and only a small fraction of tumors arising in the sacrococcygeal region. Because of its rarity, the true incidence remains unknown, and available epidemiological data are derived almost exclusively from isolated case reports and small retrospective series, mostly including adult patients.

The age distribution is bimodal, with a first peak occurring during childhood and adolescence, usually in children under 10 years of age, and a second peak in young adults between 30 and 40 years, with a slight male predominance, at least in adults [6]. Pediatric reports do not show a clear gender-related risk.

Extraspinal ependymomas most commonly arise in the sacrococcygeal region, either in subcutaneous or presacral locations, whereas rarer sites include the posterior mediastinum, the ovary and uterine ligaments [6,8].

### 3.4. Pathogenesis

The pathogenesis of ESE continues to be a point of debate. Unlike conventional ependymomas, extraspinal lesions develop outside the neuraxis and therefore require alternative embryological explanations other than the cells lining the ventricles and the spinal cord canal.

The most widely accepted hypothesis indicates that these tumors originate from ectopic ependymal cell rests derived from remnants of the caudal neural tube at the sacrococcygeal level, ectopic ependymal cells of the filum terminale, or primordial germ cells with neuroectodermal differentiation [42].

During embryogenesis, incomplete regression of the caudal cell mass may leave residual ependymal tissue within the sacrococcygeal region. These ectopic cellular remnants may subsequently undergo neoplastic transformation, giving rise to extraspinal ependymoma.

The frequent occurrence of these lesions in proximity to the coccyx supports the hypothesis that the persistence of ependymal-lined structures within the coccygeal ligament and adjacent tissues may be at the origin of these masses.

Instead, direct evidence supporting the role of primordial germ cells with neuroectodermal differentiation remains limited.

### 3.5. Classification

According to the 2021 World Health Organization (WHO) Classification of Tumors of the Central Nervous System [43], myxopapillary ependymoma has been reclassified from WHO grade 1 to WHO grade 2 because of its recognized propensity for local recurrence and metastatic dissemination, despite its relatively indolent histological appearance [44]. This change was prompted by evidence that its risk of recurrence is comparable to that of conventional spinal ependymoma, already classified as grade II [44].

### 3.6. Clinical Presentation

The clinical presentation of ESE is highly variable and largely depends on tumor size, growth pattern, and anatomical location. One of the most distinctive characteristics of this neoplasm is its indolent clinical course: most patients present with a slowly enlarging mass that has often been present for months before medical attention is sought.

In pediatric patients, the most common presentation is a painless subcutaneous swelling located in the intergluteal cleft or immediately adjacent to the coccyx, typically soft, mobile, and well circumscribed at physical examination.

Pain is reported less frequently and usually occurs when the tumor reaches a substantial size or becomes secondarily inflamed. Discomfort while sitting, local tenderness, or intermittent sacrococcygeal pain are often reported in these instances.

Chronic drainage through a sinus tract has also been documented, particularly in cases initially diagnosed as pilonidal disease.

### 3.7. Diagnostic Pitfalls and Differential Diagnosis

One of the most important findings emerging from the published literature is the high frequency of initial misdiagnosis. Because ESE typically presents as a superficial, slowly growing, and apparently benign lesion, its true identity is rarely suspected before surgery.

The rarity of the disease itself constitutes a major diagnostic obstacle. Most pediatric surgeons, general surgeons, dermatologists, and primary care physicians will never encounter a case during their clinical practice. Consequently, ependymoma is often absent from the initial differential diagnosis.

The differential diagnosis is broad and includes developmental, inflammatory, and neoplastic conditions. The most common incorrect diagnosis reported in pediatric and adult series is pilonidal disease [45] and, because of that, many patients undergo incision and drainage procedures or incomplete excisions.

Other frequent preoperative diagnoses include epidermoid cyst, sebaceous cyst, dermoid cyst, lipoma, chronic sinus tract, tailgut cyst, and presacral developmental cyst.

A 2004 study examining 15 years of experience from a tertiary pediatric pathology department on sacrococcygeal tumors emphasized several important epidemiological and diagnostic considerations [25]. Rao et al. [25] demonstrated that, although sacrococcygeal germ cell tumors represent by far the most common entity in this anatomical region in childhood, a spectrum of other rare tumors—many of which may be malignant—can also occur. Importantly, non-germ cell tumors should be considered particularly in children older than 3 years presenting with apparently new sacrococcygeal masses, since germ cell tumors rarely present beyond this age. These observations point to the diagnostic complexity of sacrococcygeal masses and reinforce the need to retain a broad differential diagnosis in older children and in atypical presentations.

Diagnostic delays reported in the literature range from several months to many years.

### 3.8. Pathology and Immunohistochemistry

Grossly, these tumors are typically well circumscribed or encapsulated, with a soft consistency and a gelatinous appearance; their color ranges from pink to gray-brown. Cystic changes and hemorrhagic events are common, often associated with secondary hemosiderin deposition and fibrotic changes.

**Figure 2:**
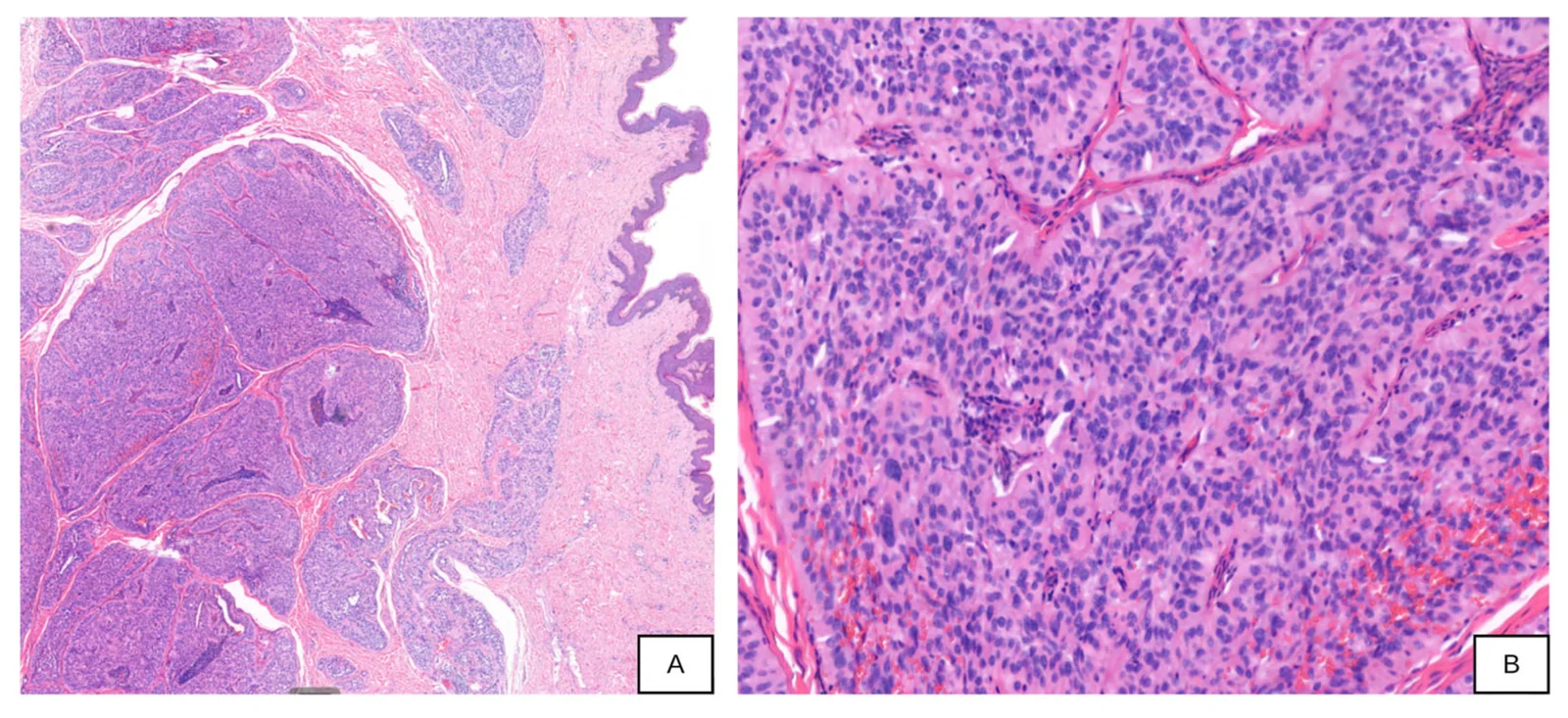
Histology of the extraspinal subcutaneous ependymoma (ESSCE): the multi-nodular dermo-epidermal neoplasia is composed of uniform epithelioid cells forming pseudo-rosettes or confluent nodules (hematoxylin and eosin: original magnification: (**A**) 2×, (**B**) 10×).

Microscopically, the majority of subcutaneous sacrococcygeal ependymomas show myxopapillary histology, with a characteristic papillary architecture: neoplastic cells, cuboidal to elongated in shape and featuring relatively monomorphic nuclei with eosinophilic cytoplasm, are arranged around delicate hyalinized fibrovascular cores. Within these structures, myxohyaline material is frequently observed; additionally, basophilic PAS-positive myxoid material can be identified in both intratumoral microcysts and perivascular spaces. These histological features are considered prototypical and, in most cases, allow for a straightforward diagnosis without the need for further ancillary studies (Figure 2).

Proliferative activity is usually low, with absent or rare mitotic figures and a Ki-67 index usually below 2–3%. However, in some cases, features associated with more aggressive behavior may be observed. In particular, the anaplastic variant of the myxopapillary ependymoma (ME) is defined by the presence of at least two of the following criteria: mitotic index greater than 2 mitoses/mm^2^, Ki-67 ≥ 10%, microvascular proliferation, and tumor necrosis [8,44].

**Figure 3:**
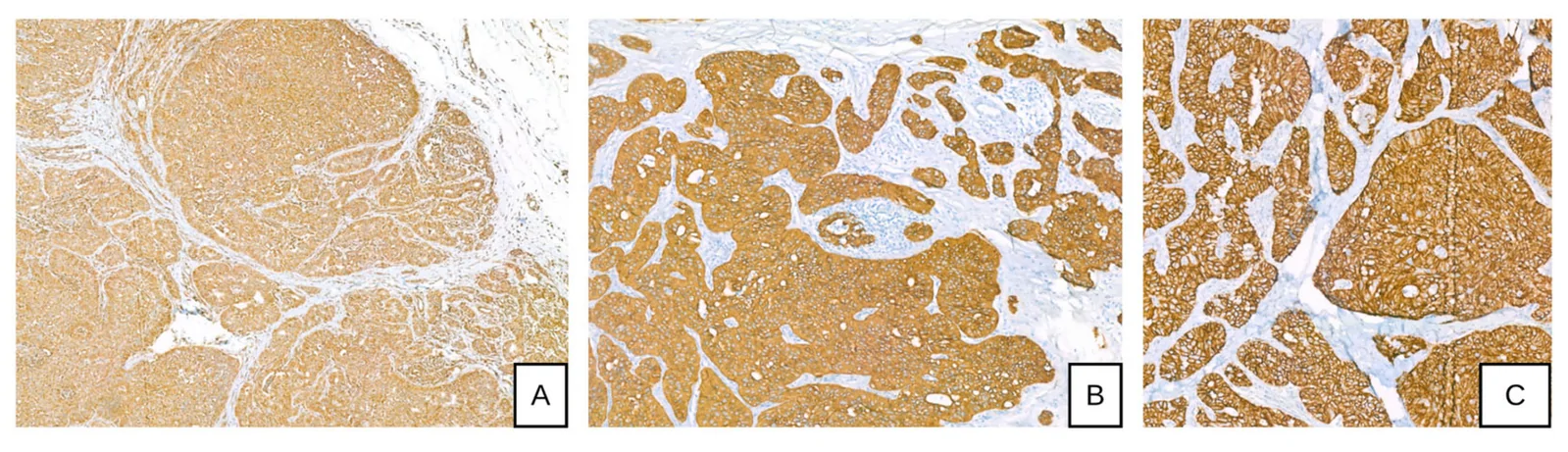
Immunoreactivity pattern of the ESSCE. The neoplasia shows intense and diffuse immunostaining for Vimentin (**A**), GFAP (**B**), and CD56 (**C**). Immunoperoxidase staining with hematoxylin counterstaining (original magnification: (**A**–**C**) 10×).

From an immunohistochemical standpoint, tumor cells typically show positivity for GFAP and S100 protein and frequently express CD99, CD56, and, in many cases, pancytokeratins (AE1/AE3). In contrast, they are generally negative for OLIG2, CAM5.2, CK5/6, CK7, and CK20. A distinguishing feature compared with other ependymoma variants is the absence of the characteristic dot-like positivity for epithelial membrane antigen (Figure 3).

Due to the diversity of its morphological features, the differential diagnosis of ME is broad and includes several neoplasms, such as metastatic carcinomas, paragangliomas, schwannomas, chordomas, and myxoid chondrosarcomas. In this context, immunohistochemistry has a key role in establishing the correct diagnosis.

Finally, recent molecular studies have demonstrated that DNA methylation profiling can distinguish ME from other ependymoma subtypes, providing an additional level of diagnostic reliability and adding to a more precise biological classification of the tumor [43,46].

The pathological differential diagnosis includes chordoma, metastatic adenocarcinoma, tailgut cyst-associated neoplasms, and other myxoid soft tissue tumors. Correlation between histopathological findings, immunophenotype, and imaging studies is therefore important for accurate diagnosis.

The distinction from chordoma is particularly relevant in sacrococcygeal lesions, because both tumors may arise in the same anatomical region and may show abundant myxoid extracellular matrix. However, their morphology and immunophenotype are usually sufficiently different to allow a reliable diagnosis. Chordoma typically shows a lobulated growth pattern composed of cords, nests, and sheets of epithelioid cells embedded in a myxoid stroma, with characteristic physaliphorous cells containing bubbly or vacuolated cytoplasm. By contrast, ME is characterized by a papillary or pseudopapillary architecture, with cuboidal to elongated tumor cells radially arranged around hyalinized fibrovascular cores and associated with myxohyaline material and perivascular mucin deposition.

Immunohistochemistry has a central role in this differential diagnosis. Chordoma usually shows diffuse expression of brachyury, cytokeratins, epithelial membrane antigen, and S100 protein, reflecting notochordal differentiation. Nuclear brachyury expression is especially useful, as it represents one of the most sensitive and specific markers for chordoma. Conversely, ME usually expresses GFAP and S100 protein, with variable cytokeratin expression, while brachyury is negative. EMA, when present in ependymal tumors, may show a dot-like or ring-like pattern, although this staining pattern may be absent or less evident in the most frequent histological subtype of extraspinal ependymoma, i.e., the myxopapillary form. Therefore, a practical immunohistochemical panel including GFAP, brachyury, pancytokeratins, EMA, and S100 protein is recommended when evaluating sacrococcygeal tumors with myxoid or chordoid morphology. In this setting, the combination of papillary architecture, GFAP positivity, and lack of nuclear brachyury supports ME, whereas physaliphorous morphology with diffuse cytokeratin/EMA expression with nuclear brachyury positivity favors the diagnosis of chordoma.

As to molecular characterization, so far, the MEs have a unique DNA methylation profile, but the prognostic significance of an ME methylation profile (also in cases with unusual histopathological features) is not clarified [43]. Also, recurrent gains of chromosome 16 and losses of chromosome 10 have been documented [43].

### 3.9. Imaging and Staging

Magnetic resonance imaging (MRI) with gadolinium contrast enhancement represents the imaging modality of choice for both diagnosis and preoperative planning: it provides excellent soft tissue characterization, accurately defines tumor extent, evaluates involvement of adjacent structures, and helps distinguish subcutaneous from presacral disease [2].

Typically, extraspinal ependymomas appear as masses demonstrating heterogeneous signal intensity on T1- and T2-weighted sequences with variable contrast enhancement. Areas of cystic degeneration, hemorrhage, or myxoid change may contribute to imaging heterogeneity.

MRI is also fundamental once the diagnosis has been established for comprehensive staging: most authors recommend MRI of the entire neuraxis to exclude occult intraspinal disease and dissemination, together with thoracoabdominal imaging to evaluate distant metastases. The role of cerebrospinal fluid cytology remains less clearly defined but may be considered in selected cases.

Furthermore, MRI is also the preferred modality for postoperative surveillance because of its ability to detect local recurrence at an early stage [47].

### 3.10. Surgical Management and Margin Status

Complete *en bloc* surgical excision with negative microscopic margins remains the cornerstone of treatment, and it is one of the key factors associated with favorable outcomes cited in the literature [48]. Because many lesions are initially presumed benign, unplanned excisions with positive margins are relatively common. In such circumstances, re-excision should be considered to obtain adequate oncological clearance.

Incomplete excision has consistently been associated with higher reintervention and recurrence rates [38].

### 3.11. Role of Coccygectomy

The role of coccygectomy remains controversial. Some authors recommend routine coccygectomy, particularly when the tumor is attached to the coccyx or when embryological remnants within coccygeal structures are suspected to represent the site of origin. This strategy is based on the hypothesis that removal of the coccyx may reduce the risk of local recurrence.

Others reserve coccygectomy for cases demonstrating direct involvement of the coccyx on imaging or intraoperative assessment. In the retrieved literature, among the 13 patients who underwent coccygectomy, 11 (84.6%) remained recurrence-free, and 2 (15.4%) experienced disease recurrence. Given the rarity of the disease and the absence of comparative studies, definitive recommendations cannot be established.

Nevertheless, the available literature strongly recommends coccygectomy in those cases where there is tumor adherence to coccygeal structures or when complete oncological resection cannot otherwise be achieved [38].

### 3.12. Adjuvant Therapy

The role of radiotherapy is less clearly defined than in intracranial ependymomas: in ESE its use is yet to be delineated. Given that cases are few and far between, treatment recommendations are largely extrapolated from adult experience and extracranial localizations in general.

While chemotherapy has generally shown limited efficacy and is seldom used, radiotherapy has primarily been employed in high-risk scenarios, such as incomplete resection, positive surgical margins, recurrence, or metastatic disease. Some reports suggest improved local control following adjuvant radiation in high-risk patients. However, robust evidence supporting its routine efficacy in children is lacking, especially taking into account that, in this population, a careful balance between potential local control benefits and long-term toxicity is required.

Therefore, radiotherapy can generally be recommended in cases of subtotal resection, unresectable disease, recurrence, or metastatic spread [38,42,48,49], but it cannot be considered a standardized adjuvant treatment in pediatric ESE.

Among the cases reported in the literature, only 1 patient has undergone adjuvant therapy (3%).

Currently, complete surgical excision remains the primary therapeutic objective, while adjuvant treatments should be individualized through multidisciplinary discussion.

### 3.13. Recurrence, Metastasis and Long-Term Surveillance

Extraspinal ependymomas exhibit a more aggressive biological behavior when compared to their intraneural counterparts. They frequently present with a higher rate of metastasis at initial diagnosis: approximately 10% of pediatric cases reported in the literature present with metastases at onset, most commonly involving the inguinal lymph nodes. Furthermore, during post-treatment surveillance, these tumors carry a significant risk of local recurrence and distant metastases (including lymph nodes, lungs, bones, and liver), which may occur even 10–20 years after initial treatment, thus requiring long-term follow-up [6,8,10,50,51]. Notably, metastases may develop in the absence of local recurrence, suggesting that hematogenous dissemination can occur even in tumors with otherwise indolent histological features. Long disease-free intervals between primary treatment and recurrence have been repeatedly reported, with some adult cases recurring more than a decade after initial surgery.

It is still not clear how often and for how long surveillance is needed; however, due to the possibility of late recurrences, long-term follow-up is recommended, and it is considered mandatory [52]. While clinical presentation and surgical feasibility are directly presented in pediatric case reports, broader management recommendations are not. Although no standardized follow-up protocol currently exists and surveillance schedules vary across centers, most authors, on the basis of expert opinion and extrapolated adult data, favor periodic clinical evaluation and MRI surveillance, particularly during the first years after treatment, with continued long-term monitoring.

### 3.14. Key Points

Due to the uncommon occurrence of ESE, knowledge regarding its optimal management remains incomplete; the currently available literature consists almost exclusively of isolated case reports and small retrospective series. As a result, there are no standardized diagnostic and therapeutic guidelines, leading to significant clinical challenges, such as frequent initial misdiagnosis and heterogeneous management approaches.

Nevertheless, several clinically relevant patterns emerge consistently across the published pediatric literature:

- First of all, the tumor usually follows an indolent course, and most often presents as a slowly enlarging sacrococcygeal mass with minimal or nonspecific symptoms. This deceptively benign presentation represents one of the main diagnostic challenges; as a result, patients frequently underwent limited excision or drainage procedures before the correct diagnosis was established.
- Histology has a crucial role in the correct and definite diagnosis of this entity, then the clinical management of the patient. A thorough histological analysis, including morphology and immunohistochemistry, is required for differential diagnosis from other possible neoplastic masses in the sacrococcygeal region, first chordomas.
- These findings emphasize the significance of thorough preoperative assessment of atypical sacrococcygeal lesions. Although imaging features are not specific, magnetic resonance imaging usually supplies essential information regarding lesion extent, involvement of deeper planes, and relationship with presacral structures [47]. MRI should be strongly considered in cases of recurrent lesions, persistent drainage, atypical location, rapid growth, or failure of presumed benign disease treatment. Increased awareness among pediatric surgeons, pediatricians, radiologists, and primary care physicians may facilitate earlier diagnosis and more appropriate management [5,10,33,38].
- Third, complete surgical excision with negative margins appears to be a critical factor associated with favorable outcomes; thus, the objective of surgery is definitive oncological cleansing at first or at early reintervention [53,54,55]. Across the literature, surgery represents the mainstay of treatment [13,38].
- Finally, despite their generally slow growth and somewhat favorable histological appearance, ESE cannot be considered entirely benign. Both local recurrence and distant metastases have been reported in pediatric patients [14,23,26]. Moreover, recurrence may occur after prolonged disease-free intervals, indicating that initial complete resection does not eliminate the risk of later progression.

## 4. Limitations

Several limitations of the available literature must be acknowledged. First, because pediatric ESE is extremely uncommon, the published evidence is limited to a small number of isolated case reports and small retrospective series published over several decades. This introduces inherent publication bias, as unusual or complicated cases are more frequently reported. Additionally, the included reports exhibit significant heterogeneity in diagnostic workups, treatment approaches, and follow-up duration, frequently accompanied by missing clinical data. Finally, the lack of comparative trials precludes high-level, evidence-based management recommendations.

Nevertheless, this structured narrative review provides a valuable synthesis of the current clinical landscape for this rare tumor.

## 5. Conclusions

When ependymoma is unexpectedly identified, referral to a multidisciplinary team is recommended to ensure appropriate staging, evaluation of surgical margins, consideration of re-excision if needed, and structured long-term follow-up.

Future progress will depend on multicenter collaboration and the development of international registries capable of collecting standardized clinical, radiological, pathological, and outcome data. Such collaborative efforts would greatly aid in overcoming the limitations of current single-center experiences in order to improve understanding of tumor biology, help identify prognostic factors, and establish definitive, evidence-based recommendations for surgical management and surveillance [43].

To optimize these future multicentric frameworks, a broader consensus should ideally focus on three core pillars: the identification of relevant clinical indicators to design a standardized minimal dataset; the establishment and active sharing of a dedicated international registry among target specialists; and the subsequent formation of multidisciplinary expert panels to issue interim clinical recommendations based on pooled data, thereby reducing current practice heterogeneity.

Until stronger data are available, heightened clinical suspicion and meticulous long-term follow-up remain the most effective strategies to improve outcomes in children with ESE.

## Figures and Tables

**Table 1:** Classification of pediatric presacral masses (adapted from: [2]).

Etiology	Lesion
**Congenital**	Germ cell tumors (sacrococcygeal teratomas, germinomatous and non-germinomatous germ cell tumors)
	**Anterior sacral meningocele**
	**Developmental cysts (epidermoid cysts, dermoid cysts, enteric cysts [rectal duplication cysts, tailgut cysts])**
	**Chordoma**
	**Cystic lymphangioma**
	**Lipoma**
**Inflammatory**	Inflammatory bowel disease (ulcerative colitis, Crohn’s disease)
	**Perirectal abscess**
	**Granuloma**
**Lymphoid**	Lymphoma (usually non-Hodgkin)
	**Post-transplantation lymphomatous disorder**
**Mesenchymal**	Rhabdomyosarcoma and undifferentiated sarcoma
	**Vascular mass**
	**Fibroma**
**Neurogenic**	Neuroblastoma
	**Ganglioneuroblastoma**
	**Ganglioneuroma**
	**Neurofibroma**
	**Schwannoma**
**Osteogenic**	Extension of sacral bone tumors
	**Giant cell tumors**
	**Aneurysmal bone cysts**
	**Osteoblastoma**
	**Ewing sarcoma family**
	**Osteogenic sarcoma**
**Other**	Hematoma
	**Extension or metastasis to the presacral space from another site**

**Table 2:** Review of the literature of the last 40 years about pediatric myxopapillary sacrococcygeal ependymoma (sex: F = female; M = male; N/A: not available).

Authors	Sex	Age (Years)	First-Line Treatment	Coccygectomy	Recurrence	Metastasis *
Ciraldo et al., 1986 [12]	F	0.8	Surgery	Yes	No	No
Chou et al., 1987 [13]	F	16	Surgery	No	N/A	N/A
	M	9	Surgery	No	N/A	N/A
Kramer et al., 1988 [14]	M	15	Surgery	No	Yes	No
Marc’Hadour et al., 1991 [15]	F	14	Surgery	No	No	No
Gupta et al., 1992 [16]	M	1.5	Surgery	N/A	N/A	Yes
Serour et al., 1993 [17]	M	11	Surgery	Yes	No	No
Botti et al., 1994 [18]	N/A	10	Surgery	No	No	Yes
Kline et al., 1996 [19]	F	0.7	Surgery	No	Yes	Yes
	N/A	N/A	Surgery	N/A	Yes	N/A
Sawyer et al., 1998 [20]	F	13	Surgery	No	N/A	No
Ilhan et al., 1998 [21]	M	8	Surgery	Yes	No	No
Johnson et al., 1999 [22]	M	7	Surgery	Yes	No	No
Grubnic et al., 1999 [23]	M	8	Surgery	Yes	No	N/A
Aktug et al., 2000 [24]	M	5	Surgery	Yes	No	No
Rao et al., 2002 [25]	F	1.3	Surgery	Yes	Yes	N/A
Akpolat et al., 2003 [26]	M	7	Surgery	No	No	No
Trobs et al., 2006 [27]	M	9	Surgery	Yes	No	No
Beschorner et al., 2007 [28]	M	1.2	Surgery	No	No	No
Alexiou et al., 2012 [29]	F	13	Surgery + Radiotherapy	No	No	No
Chakraborti et al., 2012 [30]	F	1	Surgery	Yes	Yes	No
Cimino et al., 2014 [31]	F	0	N/A	N/A	N/A	N/A
	M	1	N/A	N/A	N/A	N/A
	F	3	N/A	N/A	N/A	N/A
	M	8	N/A	N/A	N/A	N/A
	F	10	N/A	N/A	N/A	N/A
	F	13	N/A	N/A	N/A	N/A
	M	17	N/A	N/A	N/A	N/A
Dogan et al., 2016 [32]	F	9	Surgery	No	No	No
Amin et al., 2018 [33]	F	8	Surgery	Yes	No	N/A
Schiavello et al., 2018 [6]	F	4	Surgery	Yes	Yes	Yes
	M	16	Surgery	No	No	No
	F	7	Surgery	No	Yes	No
Rogers et al., 2018 [34]	F	12	Surgery	No	No	N/A
Gupta et al., 2020 [35]	M	9	Surgery	No	No	No
Thejeel et al., 2020 [36]	F	16	Surgery	N/A	N/A	No
Liu et al., 2021 [37]	F	9	Surgery	No	No	No
Fabozzi et al., 2021 [38]	F	9	Surgery	Yes	No	No
	M	13	Surgery	No	No	No
Del Campo et al. (2021) [39]	M	7	Surgery	No	Yes	N/A
Kim and Gu, 2023 [40]	F	16	Surgery	No	No	No
Lo Piccolo et al. 2025 [41]	M	6	Surgery	No	No	No

* Distant metastasis was evaluated as either present at initial presentation or developing during follow-up: the latter’s duration was reported in the literature only in 16 of 42 (38.1%) reviewed cases (median: 5.5 years, range: 8 months to 33 years).

**Table 3:** Review of the literature of the last 40 years about pediatric mixopapillary sacrococcygeal ependymoma: initial misdiagnosis.

Initial Misdiagnosis	Number of Cases (%)
Pilonidal cyst	9 (21%)
Sacrococcygeal teratoma	6 (14%)
Dermoid cyst	3 (7%)
Hematoma	2 (5%)
Meningocele	1 (2.4%)
Lipoma	1 (2.4%)
Neurinoma	1 (2.4%)

## Data Availability

No new data were generated or analyzed in this study. This narrative review is based exclusively on data and findings reported in previously published literature, which are cited throughout the manuscript.

## Abbreviations

The following abbreviations are used in this manuscript:

World Health Organization	WHO
Extraspinal sacrococcygeal ependymoma	ESE
Extraspinal Subcutaneous Ependymoma	ESSCE
Myxopapillary Ependymoma	ME
